# Impact of low-dose calcipotriol ointment on wound healing, pruritus and pain in patients with dystrophic epidermolysis bullosa: A randomized, double-blind, placebo-controlled trial

**DOI:** 10.1186/s13023-021-02062-2

**Published:** 2021-11-08

**Authors:** Christina Guttmann-Gruber, Josefina Piñón Hofbauer, Birgit Tockner, Victoria Reichl, Alfred Klausegger, Peter Hofbauer, Martin Wolkersdorfer, Khek-Chian Tham, Seong Soo Lim, John E. Common, Anja Diem, Katharina Ude-Schoder, Wolfgang Hitzl, Florian Lagler, Julia Reichelt, Johann W. Bauer, Roland Lang, Martin Laimer

**Affiliations:** 1 Present Address: EB House Austria, Research Program for Molecular Therapy of Genodermatoses, Department of Dermatology and Allergology, University Hospital of the Paracelsus Medical University, Salzburg, Austria; 2 Landesapotheke Salzburg, Department of Production, Hospital Pharmacy, Salzburg, Austria; 3 Skin Research Institute of Singapore, A*STAR, 8A Biomedical Grove, Immunos #06-06, Singapore, Singapore; 4 EB House Austria, Outpatient Unit, Department of Dermatology and Allergology, Paracelsus Medical University, Salzburg, Austria; 5 Research Office Biostatistics, Paracelsus Medical University, Salzburg, Austria; 6 Department of Ophthalmology and Optometry, Paracelsus Medical University Salzburg, Müllner Hauptstr. 48, 5020 Salzburg, Austria; 7 Research Program Experimental Ophthalmology and Glaucoma Research, Paracelsus Medical University, Muellner Hauptstrasse 48, 5020 Salzburg, Austria; 8 Institute for Inborn Errors of Metabolism and Department of Pediatrics, Paracelsus Medical University, Salzburg, Austria; 9 Department of Dermatology and Allergology, University Hospital Salzburg, Paracelsus Medical University, Muellner-Hauptstrasse 48, 5020 Salzburg, Austria

**Keywords:** Epidermolysis bullosa, Wound healing, Pruritus, Vitamin D3, Calcipotriol

## Abstract

**Background:**

Wound management is a critical factor when treating patients with the inherited skin fragility disease dystrophic epidermolysis bullosa (DEB). Due to genetic defects in structural proteins, skin and mucous epithelia are prone to blistering and chronic wounding upon minor trauma. Furthermore, these wounds are commonly associated with excessive pruritus and predispose to the development of life-threatening squamous cell carcinomas, underscoring the unmet need for new therapeutic options to improve wound healing in this patient cohort. Vitamin D3 is acknowledged to play an important role in wound healing by modulating different cellular processes that impact epidermal homeostasis and immune responses. In this study, we evaluate the safety and efficacy of low-dose calcipotriol, a vitamin D3 analogue, in promoting wound healing and reducing itch and pain in patients with DEB.

**Methods:**

Eligible DEB patients, aged ≥ 6 years and with a known mutation in the *COL7A1* gene, were recruited to a placebo-controlled, randomized, double blind, cross-over phase II monocentric clinical trial. Patients were required to have at least two wounds with a minimum size of 6 cm^2^ per wound. The primary objective was to evaluate efficacy of daily topical application of a 0.05 µg/g calcipotriol ointment in reducing wound size within a 4-week treatment regimen. Secondary objectives were to assess safety, as well as the impact of treatment on pruritus, pain, and bacterial wound colonization in these patients.

**Results:**

Six patients completed the clinical trial and were included into the final analysis. Topical low-dose calcipotriol treatment led to a significant reduction in wound area at day 14 compared to placebo (88.4% vs. 65.5%, *P* < 0.05). Patients also reported a significant reduction of pruritus with calcipotriol ointment compared to placebo over the entire course of the treatment as shown by itch scores of 3.16 vs 4.83 (*P* < *0.05*) and 1.83 vs 5.52 (*P* < *0.0001*) at days 14 and 28, respectively. Treatment with low-dose calcipotriol did not affect serum calcium levels and improved the species richness of the wound microbiome, albeit with no statistical significance.

**Conclusions:**

Our results show that topical treatment with low-dose calcipotriol can accelerate wound closure and significantly reduces itch, and can be considered a safe and readily-available option to improve local wound care in DEB patients.

*Trial* *Registration* EudraCT: 2016–001,967-35. Registered 28 June 2016, https://www.clinicaltrialsregister.eu/ctr-search/trial/2016-001967-35/AT

**Supplementary Information:**

The online version contains supplementary material available at 10.1186/s13023-021-02062-2.

## Background

Vitamin D3 (VD3) is a hormone that is primarily known for its role in calcium homeostasis and bone metabolism. Moreover, it is a key regulator of various cellular pathways in different tissues regulating hormone secretion, cell proliferation and differentiation, and immune function [1]. In human skin, which acts as site for VD3 synthesis, it contributes to skin homeostasis and proper wound healing, engaging tissue repair mechanisms that enable keratinocytes to respond to injury and infection [2]. In this regard, VD3 influences both, the innate and adaptive immune system, by inducing the expression of anti-microbial peptides (AMPs) such as cathelicidin to elicit a first-line immune defense against microbes [3], as well as by modulating immune cell responses and inflammation [4], respectively. Proper function of these processes are particularly important for patients with the rare genetic skin disease epidermolysis bullosa (EB) who suffer from excessive tissue fragility due to mutations in structural genes important for skin integrity. As a consequence, blisters and wounds develop upon minor friction. One of the most severe forms is dystrophic EB (DEB) which is caused by homozygous or compound heterozygous mutations in the gene *COL7A1*, disrupting the formation of functional anchoring fibrils that tether the epidermis to the underlying dermis. Chronic wounding of the skin, and repeated cycles of skin regeneration, infection, inflammation and tissue remodeling subsequently promote the development of life-threatening squamous cell carcinoma (SCC) in this patient cohort [5]. VD3 is produced in the skin upon exposure to ultraviolet B (UBV) radiation but wound bandages and reduced outdoor activities limit epidermal VD3 production in affected EB individuals. Notably, VD3 deficiency (< 20 ng/ml) is a common feature in recessive DEB despite oral supplementation [6].

**Fig. 1:**
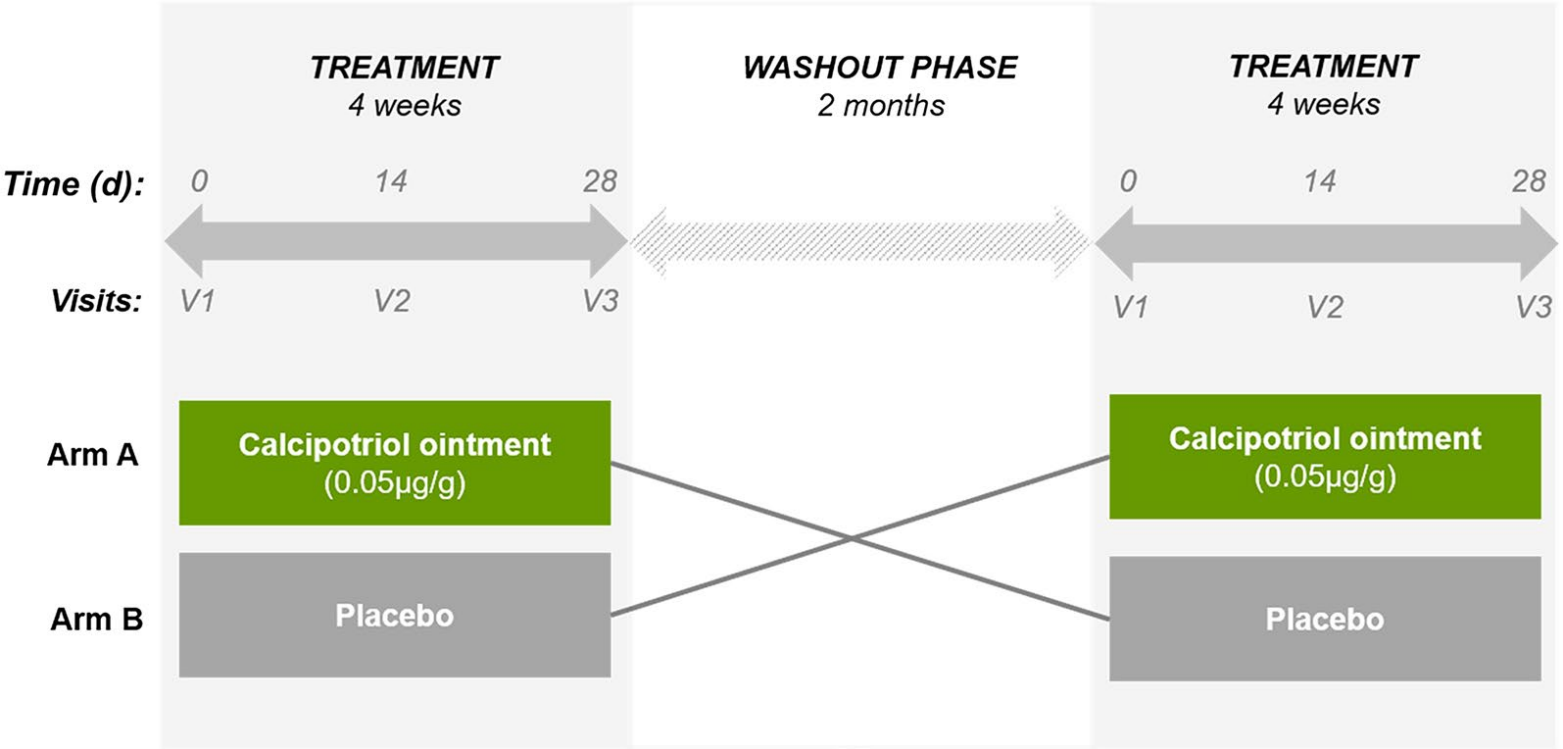
Clinical study design. We performed a two-armed, double blind, randomized, cross-over phase II study. Patients were randomized into two groups receiving either calcipotriol ointment (0.05 µg/g) or the placebo. 1 g of ointment (either verum or placebo) was applied topically on each of two designated target wounds for a period of 4 weeks. After a 2-month wash-out phase the patient crossed over into the second treatment arm. Clinical assessment of patients was performed every two weeks (day 0, 14 and 28) at the study center when wounds were photographed and swabbed for microbiome analysis

In previous in vitro investigations, we demonstrated that an induction of the AMP cathelicidin, enhanced antimicrobial defense, and accelerated wound closure in a 2D cell culture model of recessive dystrophic EB (RDEB) using a low concentration (100 nM) of the active VD3 analog calcipotriol [7]. The same treatment concentration further showed anti-neoplastic effects against established RDEB tumor cells, suggesting a safe application in this patient cohort who are highly prone to SCC development [8]. Importantly, significant clinical benefit in terms of enhanced wound closure was achieved in a single-patient observation study [7], wherein a significant reduction of itch was also reported. Together, these observations provided the rationale for evaluating the efficacy of a low-dose calcipotriol ointment in improving wound healing in RDEB patients in the present randomized, double-blind, placebo-controlled phase II clinical trial (Fig. 1).

## Results

### Patients

In total, 12 DEB patients were enrolled into the study. However, three patients did not fulfill the inclusion criteria (two wounds > 6 cm^2^) and were excluded from the analysis. A further two patients could not enter the second intervention phase because they received additional medication prohibited by the protocol during the two-month wash out phase. One patient was not included into the analysis because of incorrect follow-up of the target wounds. As a result, 6 patients were included into the final data analysis (see Flow chart, Additional file 1: Fig. 1).

### Efficacy of treatment on wound closure

**Fig. 2:**
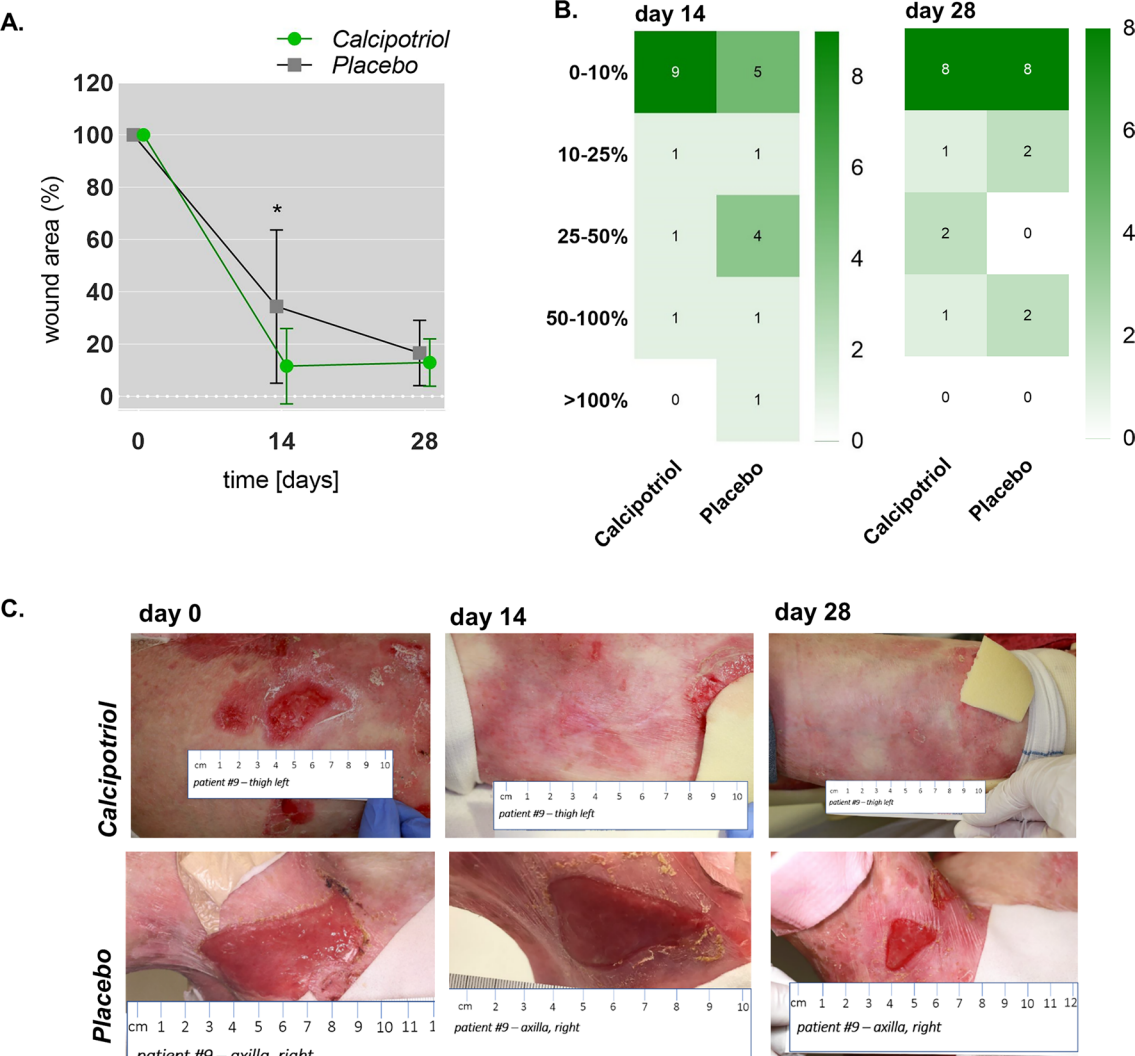
Impact of low-dose calcipotriol treatment on wound healing. **a** Size of wound area (%) representing mean with 95% confidence intervals (CIs) at baseline (day 0), 14 and 28 days of treatment with low-dose calcipotriol or placebo. Statistical analysis; generalized estimation-equation model based on gamma distribution, * *P* = 0.006. **b** Heat plot showing numbers of wounds with 0–10%, 10–20%, 25–50%, 50–100% or > 100% wound area compared to baseline after treatment with low-dose calcipotriol or placebo at 14 and 28 days. **c** Clinical pictures of wounds of a patient responding well to low-dose calcipotriol (0.05 µg/g) treatment at baseline, 14 and 28 days of treatment with low-dose calcipotriol or placebo

The clinical trial was designed as a cross-over study, wherein patients were randomized into one of two treatment arms in the first phase and, after a wash-out phase, crossed over into the other treatment arm in the second phase (Fig. 1). Accordingly, a patient who received placebo in the first phase crossed over into the calcipotriol group in the second phase, and vice-versa. In total, twenty-four wounds (12 per intervention phase) were treated equally in a ratio of 1:1 with either calcipotriol ointment or placebo (Additional file 2: Table 1). Average wound sizes at the beginning of each treatment were 16.41 cm^2^ and 15.67 cm^2^ in the calcipotriol and placebo arms, respectively (Fig. 2a and c). We observed a significant 88.4% reduction in wound size with calcipotriol treatment versus 65.6% with placebo (*P* = 0.006; generalized estimation equation model) at day 14. At that time point, calcipotriol reduced wound size to < 10% of baseline in 9/12 wounds, as compared to 5/12 which received the placebo. Both treatment and placebo resulted in the same number of wounds (8/12) with sizes < 10% of baseline after 28 days (Fig. 2b). Notably the fraction of wounds that were completely closed was higher in the calcipotriol group compared to placebo although this did not reach statistical significance (7 vs 4, 58.3% vs 33.3%; Fisher’s exact test, *P* = *0.4136*) (Additional file 3: Table 2).

### Itch and pain

**Fig. 3:**
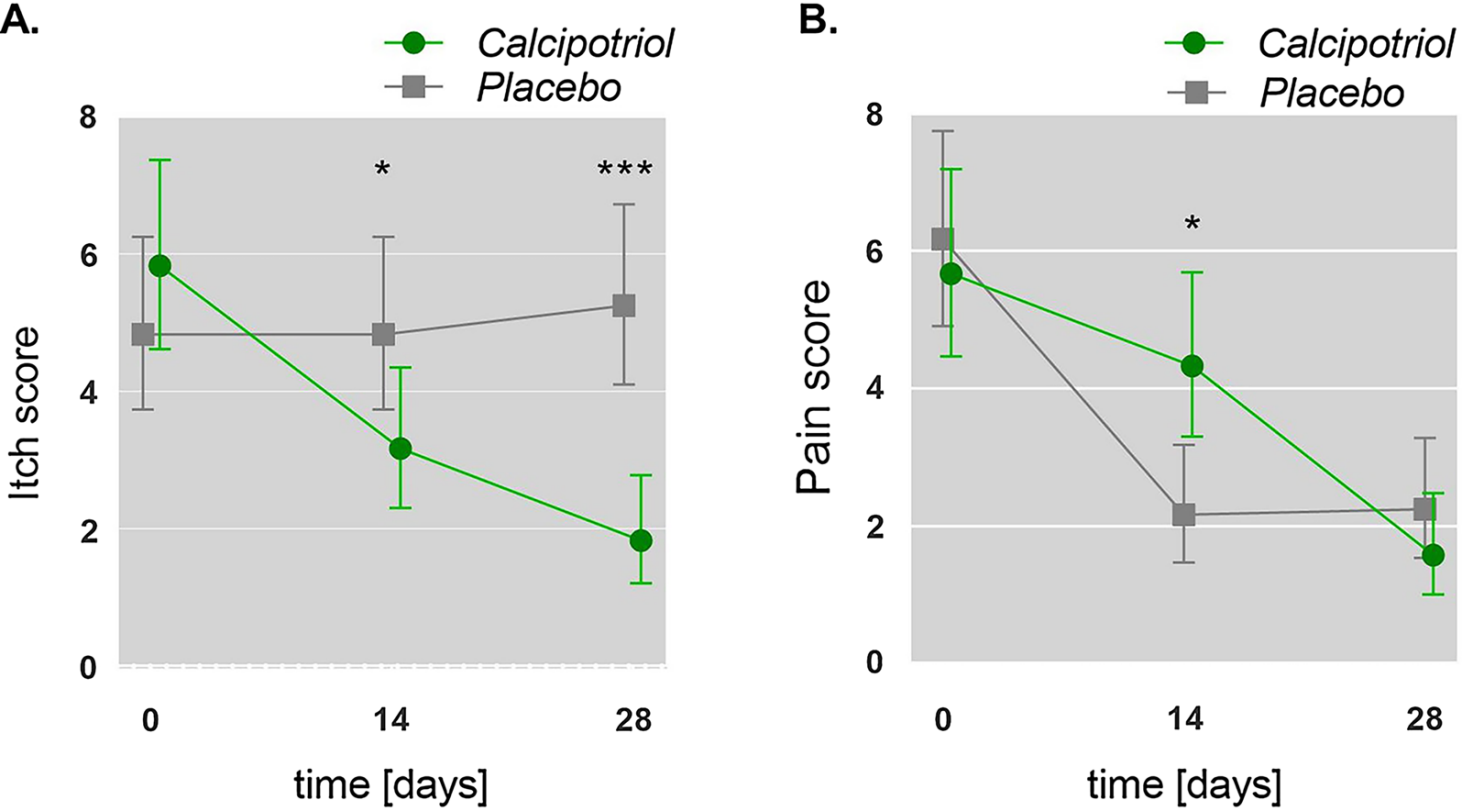
Influence of low-dose calcipotriol treatment on itch (**a**) and pain (**b**). Scores were assessed for each wound at baseline (day 0), 14 and 28 days of treatment with low-dose calcipotriol or placebo using a visual analog scale (VAS) ranging from 0 (no itch/pain) to 10 (maximum itch/pain). Mean with 95% Confidence Intervals (CIs) are shown. Statistical analysis; generalized estimation-equation model based on gamma distribution,*P < *0.05*, ***P < *0.0001*

Pruritus and pain rank high among reported patient-relevant complications associated with DEB [9]. Therefore, we assessed the impact of treatment on patient quality of life by evaluating itch and pain using a visual analog scale. Patients reported a significant and steady reduction in itch over the entire treatment course in the calcipotriol arm (Fig. 3a). No change in itch was recorded in the placebo arm. Both treatments resulted in reduced pain. While significantly improved pain scores were reported with placebo on day 14 (Fig. 3b), no significant differences between both treatment arms were observed at day 28.

### Safety assessment

No drug-related adverse reactions were observed with calcipotriol treatment. Two patients (*P03* and *P09*) gave consent for blood sampling for evaluation of serum calcium levels to exclude hypercalcemia. Neither treatment affected serum calcium levels, which remained within the reference range of 2.13 – 2.63 mmol/l at the time points tested (Additional file 4: Table 3).

### Microbiome analysis

**Fig. 4:**
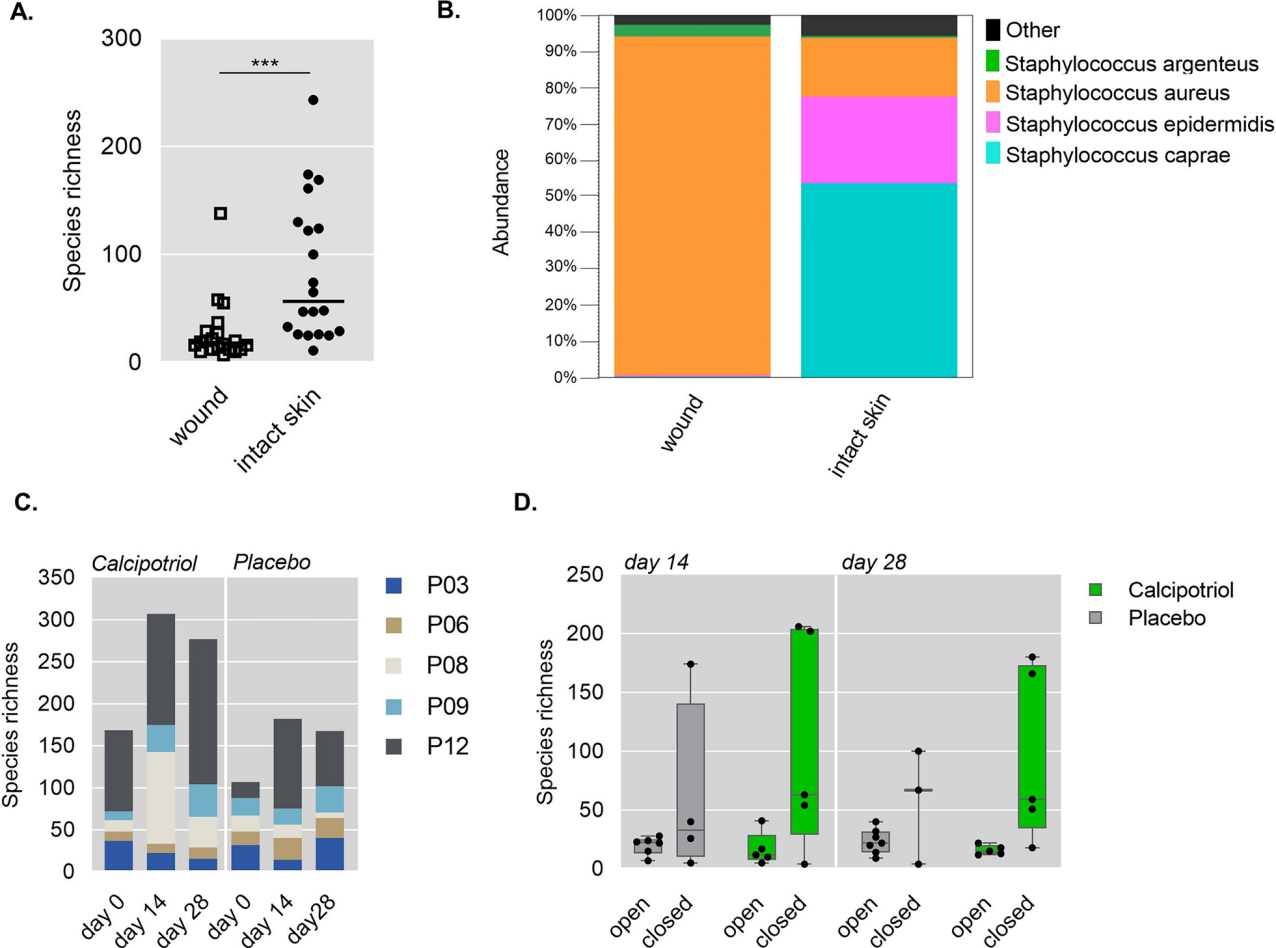
Effects of low-dose calcipotriol treatment on wound microbiome. **a** Scatter plot showing species richness on intact skin compared to wounds. Each dot represents an individual wound at baseline (day 0). Statistical analysis; Mann Whitney test, ***P = *0.0001*. **b** Relative abundance of staphylococcus species in all wounds and on intact skin at baseline (day 0). **c** Microbial species richness of wounds at baseline, after 14 and 28 day-treatment with low-dose calcipotriol or placebo. Data are presented as mean of two wounds of individual patients. **d** Species richness in open vs completely closed wounds after 14 and 28 day- treatment with low-dose calcipotriol or placebo

We evaluated the impact of treatment on wound microbiota by shotgun whole-metagenomics sequencing from 5 patients (*P03,* *P06,* *P08,* *P09,* *P12*). Wounded areas showed reduced microbial diversity and increased abundance of *Staphylococcus* *aureus* compared to control intact skin (Fig. 4a and b), confirming previous observations [4, 5]. Three out of 5 patients showed increased species richness upon calcipotriol on day 14 compared to placebo (Fig. 4c), but no general effect on *S.* *aureus* abundance was observed (data not shown). Thus, while a tendency towards improved wound microbiome was observed in calcipotriol-treated closed wounds at day 14 and day 28 compared to placebo, this did not reach statistical significance (Fig. 4d).

## Discussion

This trial suggests that topical application of calcipotriol 0.05 µg/g ointment is safe and well-tolerated by DEB patients. Topical Psorcutan® ointment (active ingredient calcipotriol 50 µg/g) is licensed for the treatment of psoriasis with an established safety profile for long-term use in adults and children (< 6 yrs). Based on previous evidence, we applied a 1000-fold less concentrated calcipotriol ointment onto the wounds of DEB patients in this present study. Both its established safety profile and the significantly reduced concentration of calcipotriol used, argued for the adequate safety standard of this approach. Nevertheless, we provide data to address safety concerns of potential systemic uptake of the drug owing to reduced skin barrier function in our patient cohort, and hypercalcemia due to VD3 regulation of calcium metabolism. Notably, monitoring for serum hypercalcemia subsequent to excessive calcipotriol uptake was restricted to study participants consenting to blood draws, reflecting the intention to limit invasive procedures on vulnerable skin in order to reduce the trial burden. By contrast, clinical symptoms of hypercalcemia (such as concentration disorder, personality change, adynamia, myasthenia, depression, dyspepsia, nausea, anorexia, constipation, polyuria, polydipsia, bone pain) were regularly assessed in all patients at each visit. Against this background, clinical monitoring remained inconspicuous in all patients and laboratory analyses of two probands additionally showed no impact of treatment on serum calcium levels.

In psoriasis, calcipotriol is used to target keratinocyte dedifferentiation and hyperproliferation. Such an antiproliferative effect would be obviously detrimental in the setting of this trial, which is intended to foster wound healing in RDEB, a process that requires cell proliferation. However, our previous studies demonstrated that lower concentrations of calcipotriol have no impact on keratinocyte proliferation in vitro [7]. In line with these observations, our clinical data showed that wound closure was accelerated over placebo in 5/6 patients after 2 weeks of treatment. Notably, accelerated wound closure in a greater fraction of wounds may translate into significant clinical benefit for patients. Accumulating evidence in mouse models highlight the interplay between microbial triggers, innate immune responses, and persistent skin inflammation in wounding-induced carcinogenesis, identifying critical molecular factors and immune cell subsets also shown to be relevant in dystrophic EB [10–12]. In this respect, the timely resolution of wounds potentially limits exposure of keratinocytes to these carcinogenic triggers.

Itch and pain rank highest among EB-associated morbidities that significantly decrease patient quality of life [9, 13]. With regard to the analgesic potential of calcipotriol, our results remain inconclusive and elusive with patients reporting a statistically significant benefit at day 14 with placebo, but no difference between placebo and verum at day 28. However, oral substitution of D3 has been suggested to be beneficial for chronic pain management in individuals with VD3 deficiency [14]. A striking effect of calcipotriol over placebo was the reduction in itch, which was reported by 5/6 patients in at least one wound and by 4/6 patients in both wounds. The anti-pruritogenic effect of calcipotriol has been previously reported in clinical trials treating patients with psoriasis [15]. It can be partly attributed to the anti-inflammatory function of VD3 that stimulates T cells to reduce the expression of inflammatory cytokines e.g. TNF-alpha, IL-1, IL-2, IL-6 or IL-8 [4]. Notably, increased levels of inflammatory cytokines are detected in blister fluids and plasma from DEB patients as well [16–18]. Calcipotriol may also indirectly improve burden of itch by inducing the AMP cathelicidin (LL-37), which primarily acts in innate immune defense against microbial infection but in turn has also been shown to induce the nerve repulsion factor semaphorin 3A [19] thereby implicating a potential anti-pruritogenic effect. However, further investigations are warranted to decipher the molecular mechanisms underlying the anti-pruritogenic effects of low-dose calcipotriol in the EB skin.

The composition of the skin microbiome is essential for overall skin health contributing to tissue homeostasis and proper wound healing. Furthermore, skin commensals have been demonstrated to be important for controlling local inflammation and tuning resident T cell function in order to provide protective immunity against cutaneous pathogens [20]. This observation is highly relevant for RDEB patients since metagenomics analysis have revealed reduced microbial diversity with a corresponding increase in pathogenic species in skin and wounds in this patient cohort [21] suggesting a potential impaired immune function and wound healing due to dysbiosis. In this trial, we observed increased species richness in 3 out of 5 patients upon 2 weeks treatment with calcipotriol, suggesting a beneficial effect on microbial diversity. Considering its antimicrobial, immunomodulatory and wound healing potential, calcipotriol may thus interfere with key pathogenic traits in EB, including tumorigenesis, which supports its implementation into local wound management strategies.

A major limitation of this study is the low patient number owing to the rareness of the disease [22], which additionally precluded the stratification of acute and chronic wounds to identify those that would benefit most from treatment. The study time points, initially chosen based on reports of EB wounds remaining open for > 4 weeks [23], appear to inadequately reflect the potency of topical low-dose calcipotriol on wound closure. Protocol-based intensified wound care may have additionally enhanced the effects of calcipotriol and placebo treatment over standard of care, potentially contributing to overall accelerated wound closure in this study compared to published literature. Thus, the primary objective, i.e. a 40% reduction of wound area with calcipotriol compared to placebo after 4 weeks treatment was not reached. In this context, our data indicate that assessment at earlier time points (< 14 days) is warranted. Time-to-wound-closure may additionally be a more relevant endpoint to comprehensively determine the therapeutic impact of calcipotriol treatment. A dose-finding study, which so far could not be performed due to limited patient numbers, may further aid in determining the optimal dose for wound healing and its impact on natural disease course.

## Conclusions

While corroboration of our results by large-scaled studies is pending, our preliminary data suggest that topical low-dose calcipotriol ointment significantly reduces itch, accelerates wound healing, and can be safely implemented into the daily wound care of DEB patients.

## Methods and study design

### Patient cohort

In total, we enrolled 12 RDEB patients with a diagnosed *COL7A1* gene mutation, of which 9 completed both intervention phases. 6 patients were included into the final analysis (Additional file 1: Fig. 1 and Additional file 2: Table 1). All patients gave written informed consent to participate in the trial. Exclusion criteria included pregnancy, breast feeding, participation in other clinical trials, known impaired kidney or liver dysfunction, known disorders of calcium metabolism, or systemic treatment with corticosteroids, immunosuppressive drugs or antibiotics.

### Study medication

Psorcutan® ointment containing 50 µg/g calcipotriol (LEO Pharma, Vienna, Austria) was diluted 1:1000 in Ultraphil® (Bayer, Vienna, Austria), reaching a therapeutic concentration of 0.05 µg/g (~ 121 nM) calcipotriol as assessed in our single-patient observation [7]. Ultraphil® base alone served as placebo control. Production and blinding of study medication was performed by the Good Manufacturing Practice (GMP)-certified drug production department of the hospital pharmacy according to EU GMP guidelines.

### Treatment

Patients were randomized into two treatment arms and instructed to apply 1 g of either verum- (active ingredient: calcipotriol 0.05 µg/g) or placebo-containing ointment topically onto two designated target wounds (≥ 6 cm^2^ each) daily over a period of four weeks. No other changes to standard of care of the patients were applied. Thus, while patients were instructed not to apply any other topical substances onto the designated target wounds, all other EB lesions were continued to be concurrently treated with the previous local wound dressings.

After a two-month wash out-phase, patients crossed over into the other treatment arm. Wounds were photographed, measured and swabbed for microbiome profiling at study visits (V) on day 0 (V1/V4), 14 (V2/V5) and 28 (V3/V6) during each treatment period.

Blood drawing was optional for the patient and was performed at the beginning and end of each treatment phase to assess serum calcium levels.

### Wound area measurement

Wound area was assessed by overlaying a transparent gridded foil onto the wound and tracing the wound edges with a permanent pen. Drawings were scanned and wound area was calculated (in cm^2^) by three independent, blinded investigators using ImageJ software (Additional file 4: Table 3). Statistical analysis was performed on mean wound area measurements. Pruritus and pain scores were assessed using a visual analog scale ranging from 0 (no itch/pain) to 10 (maximum itch/pain) at every study visit.

### Microbiome sampling and data analysis

*Sampling.* Sterile cotton swabs (Catch-All Sample collection Swab) were pre-moistened in sterile SCF-1 buffer (50 mM Tris buffer [pH 7.6], 1 mM EDTA [pH 8], 0.5% Tween 20), manufactured by the Hospital Pharmacy, Salzburg, Department of Production and immediately applied to the body site. Each target wound and the corresponding intact skin on the opposite site of the body was sampled according the Essener Kreisl technique [24] at each study visit. The head of the swab was cut off with sterile scissors, placed into sterile tubes containing SCF-1 buffer and stored at -80 °C until further processing.

### DNA extraction, library construction and sequencing

Swabs were transferred into Lysing Matrix E tubes (MP Biomedicals) together with 100 μL of ATL Buffer (Qiagen) containing 1.2% Triton X-100. Next, 10 μl of lysozyme (50 mg/ml, #90,082, Thermo Scientific) were added and incubated at 37 °C for 30 min. Samples were then subjected to bead-beating (FastPrep-24, MP Biomedicals) at 6.0 m/s for 40 s. Samples were centrifuged at 16,000 × g for 5 min. Supernatants were then treated with 10 μl Proteinase K (Qiagen) and incubated at 56 °C for 15 min. DNA was extracted (EZ1 Advanced XL Instrument, Qiagen) using the EZ1 DNA Tissue Kit (Qiagen) and quantified with Qubit dsDNA HS Assay Kit (Life Technologies) and stored at − 20 °C prior to downstream use. The extracted DNA samples were used to construct libraries using NEBNext Ultra II FS (New England Biolabs) and barcoded adaptors according to the manufacturer’s E7805 protocol. Custom indexed primers with 14 PCR cycles were used for enrichment of DNA libraries, which were quantified using Agilent DNA 1000 Kit (Agilent Technologies) and Agilent 2100 Bioanalyzer (Agilent Technologies). Samples were normalized and pooled for paired-end sequencing (2 × 150 bp) using Illumina HiSeq X platform.

### Bioinformatics analysis

The raw reads in fastq format were imported into the commercial CLC Genomics Workbench 12.0.3 using the default setting: Illumina high-throughput sequencing import, paired reads, paired-end (forward-reverse) with a minimum distance of 1 and a maximum distance of 1000. Using the default workflow of data QC and taxonomic profiling, the trimming quality score limit was set at 0.05, a trim adapter list containing Illumina adapter sequences (first read: AGATCGGAAGAGCACACGTCTGAACTCCAGTCA; second read: AGATCGGAAGAGCGTCGTGTAGGGAAAGAGTGT) for 3’ end trimming was added, a CLC curated reference database that contains 979 archaea sequences and 34,866 bacteria sequences (version June 2019 – 22 GB) was used for taxonomic mapping and the hg38 genome reference was used for host read filtering.

### Statistical analysis

Data were checked for consistency and normality. Generalized estimating equation models based on gamma and Poisson distributions were used. Least significant difference tests were used for pairwise comparisons. All reported tests were two-sided, and *P* values < 0.05 were considered statistically significant. All statistical analyses were performed by using PASW 24 (IBM SPSS Statistics for Windows, Version 21.0, Armonk, NY) and GraphPad Prism (GraphPad Software, Inc.,Version 9.0, San Diego, CA).

## Supplementary Information

**Additional file 1: Fig. 1.** Flow diagram for crossover study.

**Additional file 2: Table 1.** Information on patients.

**Additional file 3: Table 2.** Wound areas.

**Additional file 4: Table 3.** Serum calcium levels.

## Data Availability

The dataset used and/or analyzed during the current study are available from the corresponding author on reasonable request.

## Abbreviations

EB: Epidermolysis bullosa
DEB: Dystrophic EB
RDEB: Recessive DEB
V: Visit
VAS: Visual Analog Scale
VD3: Vitamin D3
