# Supplementary material for: Impact of low-dose calcipotriol ointment on wound healing, pruritus and pain in patients with dystrophic epidermolysis bullosa: A randomized, double-blind, placebo-controlled trial
Source: Orphanet J Rare Dis. 2021 Nov 8;16:473. doi: 10.1186/s13023-021-02062-2 (PMC8576995; doi:10.1186/s13023-021-02062-2)
Supplement: Supplementary file 1 — Additional file 1: Fig. 1. Flow diagram for crossover study. [file 13023_2021_2062_MOESM1_ESM.pdf]

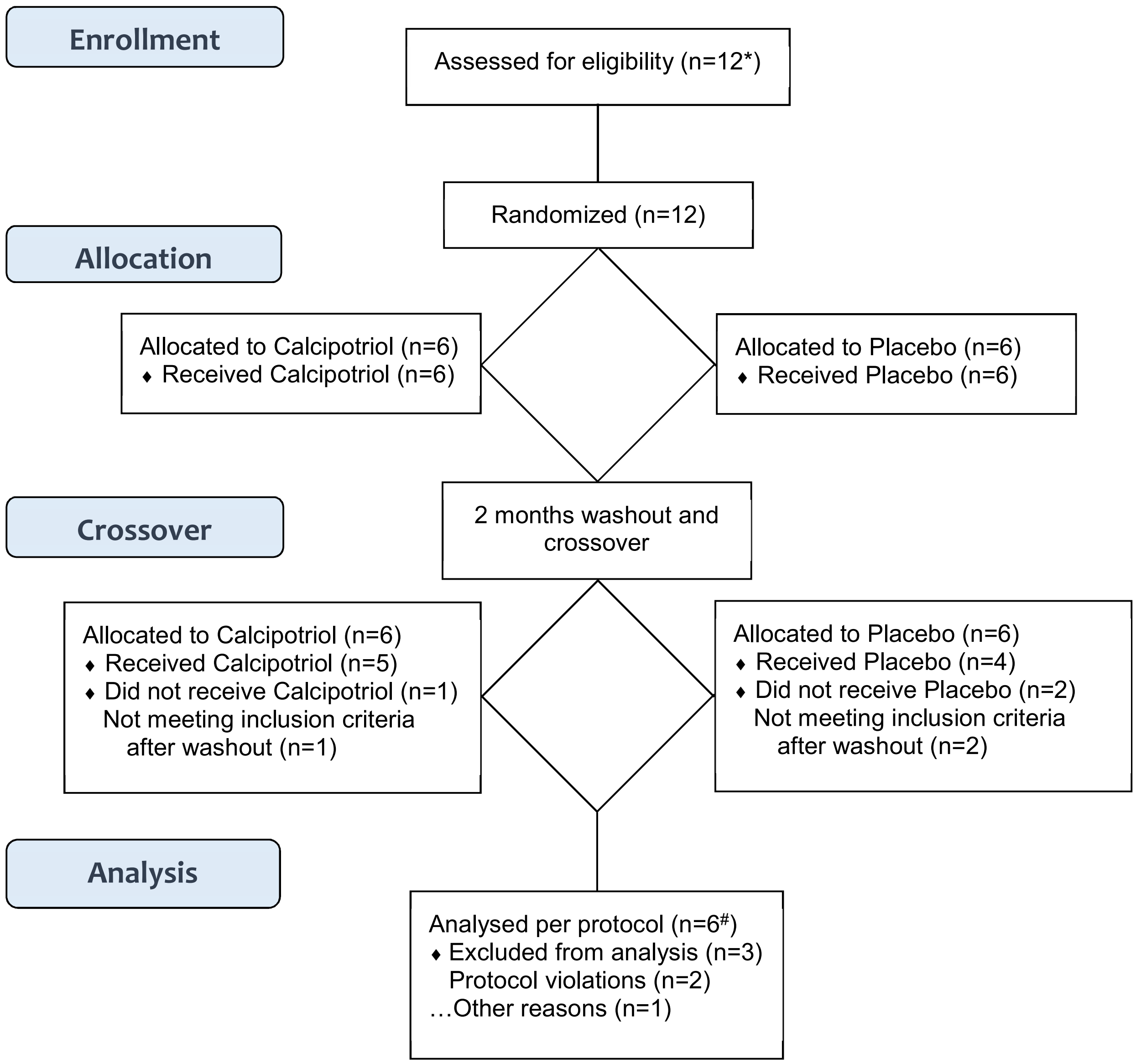

Supplementary Figure 1: Flow diagram for crossover study
* Intention-to treat –population was 15.
#5 patients were included into the analysis of the wound microbiome.

.
