# Supplementary material for: Impact of low-dose calcipotriol ointment on wound healing, pruritus and pain in patients with dystrophic epidermolysis bullosa: A randomized, double-blind, placebo-controlled trial
Source: Orphanet J Rare Dis. 2021 Nov 8;16:473. doi: 10.1186/s13023-021-02062-2 (PMC8576995; doi:10.1186/s13023-021-02062-2)
Supplement: Supplementary file 2 — Additional file 2: Table 1. Information on patients. [file 13023_2021_2062_MOESM2_ESM.pdf]

Supplementary Table 1: Information on patients, including sex, age, EB subtype and *COL7A1* mutation. In addition wound localization on the body for each intervention phase (calcipotriol and placebo) is given.

Patient Information						1st Intervention			2nd Intervention		
				***COL7A1* mutation**			***Localization***			***Localization***	
Patient ID	***sex***	***age***	***EB subtype***	***DNA***	***Protein***	***Treatment***	***Wound #1***	***Wound #2***	***Treatment***	***Wound #1***	***Wound #2***
P01	male	13	severe RDEB	c.6994C>T /c.8053C>T	p.Arg2332X / p.Arg2685X	*Placebo*	upper thigh, right	upper thigh, left	*Calcipotriol*	upper thigh, left	upper thigh, right
P03	male	33	severe RDEB	c.8440C>T/c.8440C>T	p.Arg2814X / p.Arg2814X	*Calcipotriol*	lower arm, right	lower arm, left	*Placebo*	upper arm, left	lower arm, left
P06	female	24	severe RDEB	c.6081delC / c.6081delC	p.Pro2029LeufsX177/ p.Pro2029LeufsX177	*Placebo*	shoulder, right	upper arm, left	*Calcipotriol*	shoulder, left	tibia, right
P08	female	11	severe RDEB	c.4119+1G>C / c.8523del14	splice mutation/ Glu2841Aspfs*3	*Placebo*	armpit, left	thorax lateral, right	*Calcipotriol*	knee, right	wrist, right
P09	female	31	intermediate RDEB	c.425A>G / c.6022C>T	p.Lys142Arg/ p.Arg2008Cys	*Calcipotriol*	thigh exterior, right	thigh exterior, left	*Placebo*	axilla, right	mamma, left
P12	female	7	severe RDEB	c.427-2A>G / 4172-4173insC	splice mutation/ p.1391fs*10	*Calcipotriol*	femur interior, right	upper arm, left	*Placebo*	malleolus lateralis, left	sacroiliac joint, left
