# Supplementary material for: Impact of low-dose calcipotriol ointment on wound healing, pruritus and pain in patients with dystrophic epidermolysis bullosa: A randomized, double-blind, placebo-controlled trial
Source: Orphanet J Rare Dis. 2021 Nov 8;16:473. doi: 10.1186/s13023-021-02062-2 (PMC8576995; doi:10.1186/s13023-021-02062-2)
Supplement: Supplementary file 3 — Additional file 3: Table 2. Wound areas. [file 13023_2021_2062_MOESM3_ESM.pdf]

Supplementary Table 2: Wound area (cm²) measured at each study visit during intervention phase (calcipotriol and placebo).

Wound area [cm²]													
		**P01**		**P03**		**P06**		**P08**		**P09**		**P12**	
		Calcipotriol	*Placebo*	*Calcipotriol*	*Placebo*	*Calcipotriol*	*Placebo*	*Calcipotriol*	*Placebo*	*Calcipotriol*	*Placebo*	*Calcipotriol*	*Placebo*
day0	#1	15,18	8,98	10,81	11,37	43,50	11,12	19,03	20,89	10,60	24,67	10,75	17,46
	#2	7,57	11,97	6,88	15,03	27,84	19,99	16,72	6,67	16,78	15,93	11,22	23,93
day14	#1	0,97	3,49	0,00	0,91	32,45	13,52	0,67	0,00	2,06	10,33	0,00	0,00
	#2	0,00	2,40	0,46	5,74	7,88	18,81	0,00	0,00	0,00	7,82	0,00	0,00
day28	#1	0,00	7,34	6,24	0,00	18,22	0,88	2,48	0,33	4,40	4,40	0,00	0,20
	#2	0,00	0,00	0,00	10,16	0,37	1,84	0,00	0,00	0,00	1,82	0,00	0,00
