# Supplementary material for: Impact of low-dose calcipotriol ointment on wound healing, pruritus and pain in patients with dystrophic epidermolysis bullosa: A randomized, double-blind, placebo-controlled trial
Source: Orphanet J Rare Dis. 2021 Nov 8;16:473. doi: 10.1186/s13023-021-02062-2 (PMC8576995; doi:10.1186/s13023-021-02062-2)
Supplement: Supplementary file 4 — Additional file 4: Table 3. Serum calcium levels. [file 13023_2021_2062_MOESM4_ESM.pdf]

Supplementary Table 3: Serum calcium concentration levels (mmol/l) were assessed in patients P03 and P09 before and after intervention with calcipotriol or placebo.

Patient #	**Calcipotriol**		**Placebo**	
	V1	*V3*	*V4*	*V6*
P03	2.4	2.27	2.29	2.39
P09	2.34	2.37	2.22	2.29
	Reference 2.13 – 2.63 mmol/l			
